# Self-reported aural symptoms, headache and temporomandibular disorders in Japanese young adults

**DOI:** 10.1186/1471-2474-14-58

**Published:** 2013-02-06

**Authors:** Rahena Akhter, Manabu Morita, Disuke Ekuni, Nur Mohammad Monsur Hassan, Michiko Furuta, Reiko Yamanaka, Yoshizo Matsuka, David Wilson

**Affiliations:** 1 School of Dentistry and Health Sciences, Charles Sturt University, Po Box 883, Leeds Parade, Orange, NSW, 2800, Australia; 2 Faculty of Dentistry, Professorial Unit (Jaw Function and Orofacial Pain), Westmead Centre for Oral Health, Westmead Hospital, Westmead, NSW, 2145, Australia; 3 Department of Preventive Dentistry, Okayama University Graduate School of Medicine, Dentistry and Pharmaceutical Sciences, 2-5-1 Shikata-cho, Okayama, 700-8525, Japan; 4 Sydney Medical School, The University of Sydney, Westmead, NSW, 2145, Australia; 5 Department of Fixed Prosthodontics, Institute of Health Biosciences, The University of Tokushima Graduate School, 3-18-15 Kuramoto-cho, Tokushima, 770-8504, Japan

**Keywords:** Headache, Temporomandibular disorders, Tinnitus, Vertigo, Young adults

## Abstract

**Background:**

To investigate the associations of aural symptoms, headache and depression with the presence of temporomandibular disorder (TMD) symptoms in a young adult population in Japan.

**Methods:**

A personal interview survey was conducted on first-year university students (n = 1,930) regarding symptoms of TMD, aural problems, headache, shoulder pain and depression. Logistic regression was applied to assess the associations of these problems with the presence of TMD symptoms after controlling for age and gender.

**Results:**

Among the 1,930 students, 543 students exhibited TMD symptoms and were classified into 7 groups: clicking only (Group I, n = 319), pain in the TMJ only (Group II, n = 21), difficulty in mouth opening only (Group III, n = 18), clicking and pain (Group IV, n = 29), clicking and difficulty in mouth opening (Group V, n = 48), difficulty in mouth opening and pain (Group VI, n = 11), and combination of three symptoms (Group VII, n = 97). The control group (n = 1,387) were subjects without any TMD symptoms. After adjusting for age and gender, a strong association was observed between TMD symptoms (Group II and IV) and tinnitus (OR = 12.1 and 13.2, respectively). TMD symptoms (Group I, II and III) were also associated with vertigo and headache. Otalgia and depression were significantly associated with the presence of clicking only.

**Conclusions:**

TMD symptoms were significantly correlated to aural symptoms and headache. A functional evaluation of the stomatognathic system should be considered in subjects with unexplained aural symptoms and headache.

## Background

Temporomandibular disorders (TMDs) are characterized by various signs and symptoms including pain and dysfunction in the temporomandibular joint (TMJ) and/or the masticatory musculature. Apart from these sites of pain and dysfunction other areas of the face and neck can be involved, e.g. the temporal, occipital, and frontal areas of the head, and auricular area [1,2]. In addition, depression can be observed [3].

Aural symptoms have been implicated in the predisposition, initiation, and perpetuation of TMD but with little scientific evidence [4,5]. The most commonly reported aural symptoms in TMD patients are otalgia, tinnitus, vertigo and subjective hearing loss [6-8]. The prevalence of tinnitus in the TMD population appears to be greater than that found in the general population. The prevalence of tinnitus among patients attending TMD clinics has been reported to vary from 33% to 76% [2,5,9]. Vertigo or dizziness is a false sensation of movement or spinning or whirling motion and it occurs when the body’s equilibrium is upset. The prevalence of vertigo in TMD patients is reported to range from 40% to 70% [2]. It is well documented that TMD patients with tinnitus and vertigo have higher pain and dysfunction scores than do TMD patients without otologic symptoms [9,10].

Otalgia is also a very common symptom of functional disturbances of the masticatory system [5]. The frequency of otalgia in patients with TMD reported 5% to 42% [10]. Kitsoulis found that 10.8% of TMD subjects reported ear pain, and that 75% of those TMD subjects had severe TMD [11]. The association between aural symptoms and TMD symptoms has been variously related to the position of the mandible and TMJ, retrusion of the condyles and spasm of the tensor tympani and stapedius muscles [12]. Other evidence has suggested an anatomical link between the TMJ and the middle ear through the mandibular-malleolar or diskomalleolar ligament, and neuromuscular interrelationships between the TMJ and the middle ear [12,13]. These studies on the relationship between aural symptoms and TMD have been mainly patient-based studies. However, few population studies have been published concerning the relationship between aural symptoms and TMD in adolescents or middle aged population [1,6,10]. To date, few studies have focused on young adults.

TMD is classified as a subset of primary headache disorders by the International Headache Society [14]. Findings from epidemiological and experimental-intervention studies indicate that TMD is a chronic pain condition that can occur in association with some other common chronic pain conditions, notably headaches, pain in the neck and shoulder area and back pain. Headache is regarded as the most common symptom (22%) of TMD patients, while 55% of chronic headache patients referred to a neurologist had signs or symptoms of TMD [11,15]. Investigations evaluating associations between headache and TMD could therefore be of practical relevance with both clinical and social implications. However, any hypothetical causal relationship remains difficult to establish. Further, because few population-based studies have been performed, little is known about the epidemiology of these relationships in the general population.

Depression is the psychological mood characterized by feelings of sadness, helplessness, hopelessness, guilt, despair, and futility and which have been implicated by some in the initiation of TMD [3]. Although there is no consensus regarding the percentage of TMD patients in whom depression play a role, it is clear that this possibility needs to be taken into account to properly diagnose and plan management strategies.

We hypothesize that young adult subjects having a history of aural symptoms, headache and depression there will be an association with the presence of temporomandibular disorder (TMD) symptoms.

Therefore, the objective of this study was to investigate the associations of aural symptoms, headache and depression with the presence of temporomandibular disorder (TMD) symptoms in a young adult population in Japan.

## Methods

### Study subjects

First year university students (n = 2,459) were participants in the general and oral examinations at the Health and Medical Center of Okayama University in April, 2008. Health screening is mandatory for first year students and has been routinely conducted by the Health Service Centre of Okayama University. The health screen or general health check-up comprised eye and ear check up, ECG, blood and urine routine investigation, chest X-ray etc. and dental check up. Since the health examination was mandatory, no sampling procedure was performed. Before the dental examination each subject completed a self-administered questionnaire regarding experiences of tinnitus, otalgia, vertigo, headache, depression, shoulder pain, and symptoms of TMD. Two experienced dentists obtained verbal consent and supervised the students during completion of the questionnaire to ensure that all questions were correctly understood and fully answered.

Exclusion criteria were: a previous or current smoking habit, pregnancy, any systemic diseases, consumption of any drugs within the previous 2 months, or failure to provide written informed consent. Participants over the age of 21 years were also excluded, since more than a few years had elapsed since they graduated from high school. Students who met the exclusion criteria (N = 348) or who provided incomplete data (N = 181), were excluded from the study. A total of 1,930 subjects (981 males and 949 females, respectively; 18.6 ± 2.1 years old, mean ± S.D.) satisfied the inclusion criteria and were analyzed.

### Ethical considerations

The study was approved by the Ethics Committee of Okayama University Graduate School of Medicine, Dentistry and Pharmaceutical Sciences. Ethical considerations include anonymity, privacy and ensuring informed consent was obtained. The Health Service Centre was informed in advance that the results of the examination would be published without identifying individuals. After verbal consent was obtained from all the participants during the examination period, a questionnaire and oral examination were completed by the dentists, and a general examination was performed by physicians and public health nurses.

### Questionnaire

A standardized student’s health questionnaire was used for all students. This questionnaire consisted of questions relating to a range of dental, medical, and aural complaints. Before starting the study, the specific contexts of the questions was explained to all subjects and where necessary question meaning were double checked with subjects to ensure that they understood correctly. Questions to distinguish TMD-positive cases from TMD-negative cases in accordance with previous studies [15-17] were “During the past 2 months, *(i)* Have you ever noticed any sounds around your ears? (clicking), *(ii)* Have you ever felt pain around your ears while opening your mouth or chewing food? (pain in TMJ), and *(iii)* Have you ever felt difficulty in opening your mouth? (difficulty in mouth opening).” Each question was answered by selecting a description of awareness (frequently, sometimes, rarely, or never). The reliability of such questionnaires has been verified by other studies [16,17].

The occurrence of specific aural symptoms was obtained through self-report. The participants were simply asked whether they had aural complaints during the past 2 months. Patients were asked about ear noises (tinnitus: “did you hear noises in your ears or head?”) [18], and the presence of earaches (otalgia: do you experience pain in or around your ears?), and vertigo (“did you ever experience spinning or things spinning) [1,7]. The severity of aural symptoms was not estimated. Information regarding self-report of headache (“did you have headaches in the sides of your temples during the past 2 months?”) [17] was also obtained from the questionnaire, and the type of headache was not classified. Other questions included in the questionnaire were depression (“did you feel depressed during the past 2 months?”) or shoulder pain (“did you within the past 2 months have troublesome shoulder pain?”) [17]. The questions were designed to elicit a yes or no answer.

### Statistical analysis

The data were analyzed using the software package, SPSS (version 17.0, Family, SPSS Inc., Chicago, IL, USA). The subjects were arbitrarily separated into a TMD-positive group (frequently or sometimes aware of TMD symptoms) and TMD-negative group (rarely/never aware of TMD symptoms). The chi-squared test was used for comparison of two non-numerical variables, such as yes (%) answers by respondents with and without otalgia, vertigo, tinnitus, headache, depression and shoulder pain variables. An α level of 5% was the threshold for a statistically significant difference. Multiple comparisons, involving the same subjects, increase the probability that one comparison will become statistically significant. Therefore, yes (%) answers by respondents having otalgia, vertigo, tinnitus, headache, depression and shoulder pain were compared between the groups using Bonferroni correction to adjust probability [7]. For instance, when group I (clicking only) was associated with one of these 6 categories, the number of comparisons was 6 and p-values below 0.05/6 = 0.008 were considered significant. Based on the results of bivariate analysis, variables associated with TMD symptoms were selected as possible factors related to TMD. Furthermore, the strength of association between TMD symptoms and these factors was expressed as an odds ratio (OR) and a 95% confidence interval (CI) using logistic regression models. Probability levels of *P* < 0.05 were considered to be statistically significant.

## Results

The distribution of frequencies of temporomandibular disorder (TMD) symptoms is shown in Table 1. The prevalence of TMD symptoms according to age and gender is shown in Table 2. No significant differences for age and gender distribution were observed between any of TMD-positive students and TMD-negative students. Percentages of female subjects in all TMD-positive groups were higher than the TMD-negative group except for group I and group V.

**Table 1:** Frequencies of symptoms of TMD

**Group**	**TMD symptoms**	**Frequency**	**Percent (%)**
TMD-negative		1,387	71.9
TMD-positive		543	28.1
I	Clicking only	319	16.5
II	Pain in TMJ only	21	1.1
III	Difficulty in mouth opening only	18	0.9
IV	Clicking and pain in TMJ	29	1.5
V	Clicking and difficulty in mouth opening 48	2.5	
VI	Pain in TMJ and difficulty in mouth opening	11	0.6
VII	Clicking, pain in TMJ and diff. in mouth opening	97	5.0
Total		1,930	100.0

**Table 2:** Percent distributions of age and gender according to TMD symptoms-positive and TMD symptoms-negative groups

	**TMD-negative**	**TMD-positive groups**						
**Variables**	**group**	**I**	**II**	**III**	**IV**	**V**	**VI**	**VII**
Age								
18 years	78.4	82.8	71.4	72.2	79.3	81.3	72.7	71.1
19 years or more	21.6	17.2	28.6	27.8	20.7	18.8	27.3	28.9
Gender								
Male	52.1	50.2	42.9	44.4	41.4	54.2	27.3	42.3
Female	47.9	49.8	57.1	55.6	58.6	45.8	72.7	57.7

Table 3 shows the relationships of TMD symptoms to otalgia, vertigo, tinnitus, headache, shoulder pain and depression. The percentage of subjects experiencing vertigo was significantly greater in the TMD-positive groups I, II, III, IV and V than in the TMD-negative group. The percentage of subjects experiencing tinnitus was also significantly greater in the TMD-positive groups II and IV. Significant associations were observed between the percentages of subjects experiencing headache and TMD-positive groups I, II, III, IV and VII. The percentage of subjects who were depressed and had a history of otalgia was higher in group I than those in the TMD-negative group. History of shoulder pain did not show significant relation with TMD symptoms.

**Table 3:** Prevalence (%) of vertigo, tinnitus, otalgia headache, shoulder pain and depression in the TMD-negative and TMD-positive groups

		**TMD-negative**	**TMD-positive groups**						
**Variables**		**group**	**I**	**II**	**III**	**IV**	**V**	**VI**	**VII**
Tinnitus	No	94.0	94.0	52.4	94.4	44.8	95.8	90.9	95.9
	Yes	6.0	6.0	47.6**	5.6	55.2**	4.2	9.1	4.1
Vertigo	No	87.6	79.9	61.9	66.7	55.2	72.9	72.7	81.4
	Yes	12.4	20.1*	38.1**	33.3*	44.8**	27.1*	27.3	18.6
Otalgia	No	74.5	65.2	61.9	88.9	58.6	64.6	45.5	73.2
	Yes	25.5	34.8**	38.1	11.1	41.4	35.4	54.5	26.8
Headache	No	82.0	74.3	38.1	55.6	27.6	70.8	63.6	67.0
	Yes	18.0	25.7*	61.9**	44.4*	72.4**	29.2	36.4	33.0**
Depression	No	83.7	71.8	66.7	88.9	69.0	83.3	54.5	73.2
	Yes	16.3	28.2**	33.3	11.1	31.0	16.7	45.5	26.8*
Shoulder pain	No	92.4	90.3	81.0	83.3	86.2	87.5	72.7	85.6
	Yes	7.6	9.7	19.0	16.7	13.8	12.5	27.3	14.4

*: P < 0.008; **: P < 0.001 (Significantly higher than that in the TMD-negative group by the chi-square test).

When the variables were analyzed together adjusting for age and gender, the strongest association was seen between TMD symptoms and otalgia, tinnitus, vertigo and headache (Table 4). Subjects who reported symptoms of depression were significantly associated with only group I TMD- positive symptom.

**Table 4:** Results of multiple logistic regression analysis after adjustment for age and gender

**TMD symptoms**	**Significant factors (Odds ratio and 95% CI)**^**a**^				
	**Tinnitus**	**Vertigo**	**Otalgia**	**Headache**	**Depression**
Group I (Clicking)		1.55**	1.34 *	1.36 *	1.85***
		(1.12-2.15)	(1.02-1.75)	(1.01-1.83)	(1.37-2.50)
Group II (Pain in TMJ)	12.07 ***	2.77*		5.76***	
	(4.78-30.52)	(1.05-7.32)		(2.23-14.85)	
Group III (Difficulty in mouth opening)		2.94*		3.04*	
		(1.06-8.10)		(1.16-8.00)	
Group IV (Clicking and pain in TMJ)	13.22 ***			8.30***	
	(5.75-30.39)			(3.40-20.29)	
Group V (Clicking and difficulty in mouth opening)		(1.36-5.04)			
Group VI (Pain in TMJ and difficulty in mouth opening)					
Group VII (Clicking, pain in TMJ and difficulty in mouth opening)				2.04*	
				(1.29-3.21)	

*: P < 0.05, **: P < 0.01, ***: P < 0.001, adjusted by age and gender.

^a^ Reference category: no aural symptoms.

## Discussion

To the best of our knowledge, the present study is the first report to investigate an association between aural symptoms (otalgia, tinnitus and vertigo), headache, depression and TMD in a young adult Japanese population. In previous studies, vertigo and tinnitus have been reported as aural symptoms in functional disturbances of the masticatory system [9]. Several studies have also noted otologic complaints more often in subjects with TMD than in those without TMD [6,12,13]. The present epidemiological study in young adult population also confirmed the results.

The reported prevalence of otological complaints in TMD patients varies in the literature. The most relevant article to this work is a comparably controlled study of otic symptoms in TMD by Tuz *et al.*[13]. In their 155 study patients with TMD who reported having aural symptoms, the frequency of tinnitus was 59%, whereas in our study subjects, the frequency was 39%. The difference might reflect the older population in their study group (mean age, 49.1 years) who may be more likely to have aural symptoms than our younger study group (mean age, 18.6 years). Our findings showed a relatively strong correlation between tinnitus and subjects having pain in the TMJ (group II) and the combination of clicking and pain in the TMJ (group IV) after adjusting for age and gender in the logistic regression analyses. There is evidence for a link between tinnitus and pain in the TMJ [12]. Tinnitus has been associated with pain upon pressure in the masticatory muscles and the TMJ, mandibular overclosure and posterior displacement of the condyle [9,18]. Tinnitus may have a central component (as opposed to cochlear tinnitus) but can be modified both by voluntary orofacial movements (including tooth clenching) and purely sensory stimuli [1]. Some investigators have hypothesized that eustachian tube dysfunction, masticatory muscle dysfunction or reflex-sympathetic vasospasm of labyrinthine vessels occurs secondary to abnormal stimulation of autonomic nerves of the TMJ [2]. On the other hand, Toller and Juniper [19] determined no statistical difference in the results of the analysis of audiograms, tympanograms, and eustachian tube function in TMD patients compared with their control patients. A longitudinal study is warranted to verify these considerations.

Our data are consistent with previous findings suggesting there may be a link between TMD and postural imbalance leading to a dizzy. The frequency of vertigo in patients with TMD ranges from 40% to 70% in a study by Ramirez *et al.*[20], whereas in our study it was 12 to 50%. The reason for this discrepancy might be due to the different types of study subjects. Ramirez *et al.*[20] studied only a older patient population where vertigo might be more frequent, whereas our study subjects were 1st year healthy university students who might have less tendency to vertigo. It has been suggested that malpositioning of the mandibular condyle as a result of TMD could lead to eustachian tube blockage and symptoms of aural pain and vertigo [4]. Significantly more patients in a TMD group (70%) also reported vertigo than in a control group (31%) in Chole and Parker’s study [7]. A high incidence of vertigo in their subjects with TMD may relate to the possibility that underlying emotional distress may exacerbate vertigo. Parker and Chole [2] stated that TMD and vertigo are associated with emotional disorders. However, the pathogenesis of the symptom of vertigo in subjects with TMD is still unknown.

Otalgia is often considered to be a referred pain of orofacial origin, but it could be speculated that otalgia and the sensitivity of the ear canal are influenced by chemical mediators of inflammation [21] associated with the contiguous TMJ. In our study, the prevalence of otalgia in TMD subjects was 34%. The findings of the present study were consistent with other studies [2,5]. The otalgia may possibly be explained by the proximity of the temporomandibular joint and the structures of the ear. It may be the consequence of a mechanical irritation of the auriculotemporal nerve or of some interference into the petrotympanic fissure region due to an articular inflammatory-degenerative state [10]. The cause of otalgia in patients with TMD without a pathological condition in the ears or nasopharynx is explained as referred pain from the masticatory muscles or temporomandibular joints [5]. Therefore patients without infection should be referred to a dentist with stogmatognathic experience to rule out stogmatognathic causes of aural symptoms.

Patients diagnosed with painful TMDs often report having headaches [22]. The prevalence (21.9%) of headache in our population is within the range of other studies conducted on Asian Chinese (24.2%), Japanese (22.8%), European (21.9%) and northern American populations (13-21%) [23-25]. In a univariate analysis of our data, headache was associated with symptoms of TMD as a whole and this relationship remained significant also after adjustment for age and sex. For headache, we found a significant risk between subjects with and without symptoms of TMD. The high OR for group II (12.1) and IV (13.2) suggests that in the young adult population, the relationship between headache and TMD may be primarily expressed as pain in the TMJ region. Owing to the cross-sectional study design in this project no etiological conclusions can be drawn and caution should be paid because no clinical confirmation of the location of pain was available.

TMD patients report significantly more tender points upon palpation of the shoulder and neck muscles. The TMJ and the cervical spine acts as a single functional entity, which could be one of the reasons for this association [26]. There is also some evidence from a neurophysiological point of view that the extensive convergence of different types of afferent input on the trigeminal nuclei and on neuronal plasticity [27] is the reason for the association between TMD and shoulder pain. However, to establish the exact association between shoulder pain and TMD pain further research is required.

Depression causes an increase in muscular tension which spreads to the pericranium muscles and might act as a cause for TMD symptoms. Several studies have shown that many TMD patients are depressed [3,28]. This study confirms and extends previous reports addressing the association between depression and TMD populations.

All data analyzed in our study were collected from written questionnaires. It is recognized that the data relied on memory and self reporting. The authors recognize that there might have been possible incorrect answers to questions. Another limitation of our study is the absence of clinical diagnosis of TMD in the subjects. Since the clinically determined prevalence (point prevalence) might be less than the prevalence of TMD symptoms reported on the questionnaires (period prevalence), we used period prevalence as the diagnostic criterion for TMD. The results of several studies also support the validity of questionnaires for epidemiological studies on TMD symptoms [29,30]. An additional limitation was medical records were nor analyzed, nor was a standardized questionnaire for assessing depression used.

## Conclusion

In conclusion, tinnitus, vertigo and otalgia were reported more frequently in patients with symptoms of TMD than in the control populations studied. The association of these symptoms does not prove a causal relationship, but investigations of this association may improve our understanding of both TMD and the otologic symptoms of tinnitus and vertigo especially in a young adult population. The association between headache and the TMD groups suggests that headache may be a risk factor for the development of TMD, or that TMD could be a risk factor for development of headache. The findings of this study have potentially important clinical implications for the treatment and management of patients suffering from TMD and having aural symptoms, headache and depression. More population based and longitudinal studies are desirable to determine better the nature and etiology of the relationship between these symptoms and TMD.

## Competing interests

The authors declare that they have no competing interests.

## Authors’ contributions

RA conceptualized, designed, analyzed and drafted the study. DK, MF and RY were responsible for the acquisition of data. MM helped in the study design and data interpretation. RA, NMH, YM and DW have critically revised the manuscript. All authors read and approved the final manuscript.

## Pre-publication history

The pre-publication history for this paper can be accessed here:

http://www.biomedcentral.com/1471-2474/14/58/prepub
